# Complicated COVID-19 in pregnancy: a case report with severe liver and coagulation dysfunction promptly improved by delivery

**DOI:** 10.1186/s12884-020-03172-8

**Published:** 2020-09-04

**Authors:** Louise Ronnje, John-Kalle Länsberg, Olga Vikhareva, Stefan R. Hansson, Andreas Herbst, Mehreen Zaigham

**Affiliations:** 1 Department of Obstetrics & Gynaecology, Skåne University Hospital, Malmö/Lund, Sweden; 2 Department of Clinical Sciences Lund, Paediatrics/Neonatology, Lund University, Skåne University Hospital, Malmö/Lund, Sweden; 3 Department of Obstetrics & Gynaecology, Institute of Clinical Sciences Lund, Lund University and Skåne University Hospital, 205 01 Malmö/Lund, Sweden

**Keywords:** COVID-19, Coronavirus, SARS-CoV-2, Neonate, Pregnancy, Differential diagnosis, HELLP syndrome, Liver, Coagulation, Morbidity, Fever, Mortality, Obstetric management, Pandemic, Respiratory distress syndrome, Respiratory failure, Sepsis, Susceptibility, Virus

## Abstract

**Background:**

It has been proposed that pregnant women and their fetuses may be particularly at risk for poor outcomes due to the coronavirus (COVID-19) pandemic. From the few case series that are available in the literature, women with high risk pregnancies have been associated with higher morbidity. It has been suggested that pregnancy induced immune responses and cardio-vascular changes can exaggerate the course of the COVID-19 infection.

**Case presentation:**

A 26-year old Somalian woman (G2P1) presented with a nine-day history of shortness of breath, dry cough, myalgia, nausea, abdominal pain and fever. A nasopharyngeal swab returned positive for severe acute respiratory syndrome coronavirus 2 (SARS-CoV-2) infection. Her condition rapidly worsened leading to severe liver and coagulation impairment. An emergency Caesarean section was performed at gestational week 32 + 6 after which the patient made a rapid recovery. Severe COVID-19 promptly improved by the termination of the pregnancy or atypical HELLP (Hemolysis, Elevated Liver Enzymes and Low Platelet Count) exacerbated by concomitant COVID-19 infection could not be ruled out. There was no evidence of vertical transmission.

**Conclusions:**

This case adds to the growing body of evidence which raises concerns about the possible negative maternal outcomes of COVID-19 infection during pregnancy and advocates for pregnant women to be recognized as a vulnerable group during the current pandemic.

## Background

The pandemic caused by the severe acute respiratory syndrome coronavirus 2 (SARS-CoV-2) has exposed vulnerable populations to an unprecedented global health crisis. From the knowledge gained from previous human coronavirus outbreaks, it has been proposed that pregnant women and their fetuses are particularly at risk for poor outcomes [1]. The maternal and neonatal outcomes of pregnant women with COVID-19 is limited to a handful of case reports which present diverse results. Obstetricians are still learning about COVID-19 presentation and progression in pregnancy and even though the majority of pregnancies infected by COVID-19 have good outcomes, a recent systematic analysis [1] showed that up to 3% of pregnancies were associated with severe maternal morbidity. It was indicated that mothers with a complicated medical history could be at increased risk for severe outcomes. Furthermore, experts are of the opinion that the clinical recommendations for managing COVID-19 in pregnancy should be based on lessons learned from the current epidemic [2] which emphasizes the importance of presenting COVID-19 cases associated with complex clinical management.

We therefore present a case report of a young woman, pregnant in the third trimester, diagnosed with COVID-19, showing severe liver and coagulation impairment.

## Case presentation

A 26-year-old Somalian woman (Gravida 2, Para 1) who had been living in Sweden for a year presented at the Emergency Department of Skåne University Hospital in Malmö on April 17th, 2020 pregnant at 32 + 1 weeks of gestation. She was transferred to the Infectious Diseases Department with suspicion of COVID-19. A diagnostic test, based on quantitative real time polymerase chain reaction (qRT-PCR), from a nasopharyngeal swab, was positive for SARS-CoV-2.

She had recently moved from Stockholm to Malmö. In 2015 she had a normal vaginal delivery in Somalia. The patient had an appendectomy and a cholecystectomy in Somalia. Her medical history also included hypothyroidism, currently treated with 150 *u*g Levothyroxine daily. The body mass index (BMI) on admission to prenatal care was 47 kg/m^2^ with length 163 centimetres (cm), weight 126 kg (kg). Apart from the obesity, her pregnancy had been without complications. She had received an intramuscular injection of Anti-D immunoglobulin at 28 + 5 weeks of gestation since she was Rhesus D (RhD) negative and the fetus was RhD positive.

**Fig. 1:**
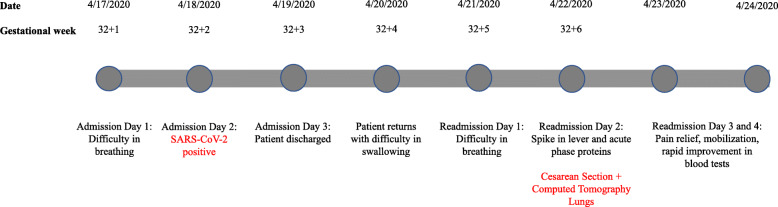
Timeline of the patient’s admission in to tertiary care

On admission, the patient described a nine-day history of shortness of breath, dry cough, myalgia, nausea, abdominal pain and fever (Fig. 1). She had significant abdominal pain on admission but the surgeon did not find any signs of an acute abdominal event. The patient had also noticed reduced fetal movements for the last two days. Obstetric examination including cardio-tocography (CTG) and an abdominal ultrasound showed no abnormalities.

The patient’s respiratory rate was 22 breaths/minute, oxygen saturation 95%, blood pressure 116/71, pulse 113 beats/minute and temperature 37.2 degrees Celsius (^o^C). The laboratory tests are shown in Table 1. The patient was given morphine, paracetamol and oxycodone for pain relief and also received thromboprophylaxis, Dalteparin 7500 units /day subcutaneously. No additional oxygen was needed.

**Table 1:** Maternal laboratory results during admission and readmission

Variable	Reference Range	Admission Day 1 (4/17/2020)	Readmission Day 2 (4/22/2020) Prior to Cesarean Section	Readmission Day 3 (4/23/2020)
Hemoglobin (Hb) g/L	117–153	113	95	101
Platelet count x10^9^/L	165–387	209	122	179
White cell count x10^9^/L	3.5–8.8	5.1	4.4	7.0
Neutrophil count x10^9^/L	1.8–7.5	2.8	3.1	6.1
Lymphocyte count x10^9^/L	1.0–4.0	1.9	0.3	0.5
Reticulocyte x10^9^/L	28–120	-	38	53
Haptoglobin g/L	0.24–1.90	-	2.02	-
Ferritin µmol/L	13–148	-	875	370
C-reactive protein (CRP) mg/L	< 5	37	102	136
Interleukin-6 ng/L	< 1.8	-	211	-
Procalcitonin µg/L	< 0.05	-	149	209
P-NT-pro-BNP ng/L	< 150	-	71	-
Troponin-T ng/L	< 5	< 5	-	-
Myoglobin µg/L	25–58	< 22	34	-
Glucose mmol/L	4.2-6.0	-	4.3	4.4
Aspartate aminotransferase (ASAT) µkat/L	0.25–0.6	0.38	28	11
Alanine aminotransferase (ALAT) µkat/L	0.15–0.75	0.84	5.8	3.5
Alkaline phosphatase (ALP) µkat/L	0.70–1.9	-	4.6	3.9
Gamma-glutamyl transferase (GGT) µkat/L	0.15–0.75	-	5.4	5.7
Bilirubin µmol/L	5–25	-	21	21
Lactate Dehydrogenase (LDH) µkat/L	1.8–3.4	-	34	9.9
Pancreatic amylase µkat/L	0.15–1.1	0.30	0.15	0.12
Creatinine µmol/L	45–90	50	59	63
Urea mmol/L	2.6-6-4	-	1.3	1.0
Uric acid µmol/L	155–350	-	312	267
Sodium mmol/L	137–145	136	135	136
Potassium mmol/L	3.5–4.4	3.5	2.8	3.6
Chloride mmol/L	98–110	-	107	103
Calcium ion mmol/L	1.15–1.33	-	1.10	1.06
Magnesium mmol/L	0.70–0.95	-	0.62	0.73
Plasma Paracetamol concentration µmol/L	< 200	-	67	-
D-dimer mg/L	< 0.5	5.4	4.3	5.6
Prothrombin-complex International Normalized Ratio (P-INR)	0.9–1.2	0.9	1.0	1.0
Activated Partial Thromboplastin Time (APTT) in seconds (s)	26–33	39	46	45
Fibrinogen g/L	2.0–4.0	-	2.7	2.2
Antithrombin (IIa) kIE/L	0.8–1.2	-	-	0.66
**Arterial blood gases**				
pH	7.35–7.45	-	7.43	7.40
Partial pressure of carbon dioxide pCO_2_ in kPa	4.6-6.0	-	4.1	5.1
Partial pressure of oxygen pO_2_ in kPa	10.0–13.0	-	8.6	9.9
Base Excess mmol/l	22–27	-	22	23
Bicarbonate HCO_3_^−^mmol/l	-3.0-3.0	-	-3.2	-1.7
Lactate mmol/L	0.5–1.6	-	1.5	0.7
Saturation of oxygen %	97–100	-	92	93

On day 2 (18/4/2020), the patient was relatively stable apart from two short episodes of fever up to 38.9 ^o^C. Due to risk for preterm labour, the patient received 12 milligrams (mg) of Betamethasone intramuscularly to aid fetal lung maturity. Daily fetal monitoring using CTG showed no signs of fetal distress.

The patient was discharged on day 3 (19/4/2020) with a planned obstetric follow-up including fetal growth assessment after recovery. She was prescribed dalteparin for four weeks.

The patient returned to the Emergency Department the next day (20/4/2020) with a sore throat and severe difficulties in swallowing. Apart from tachypnoea (25–35 breaths/minute) and tachycardia (118 beats/minute), other vital signs were normal. After examination, she was discharged with a prescription of Betamethasone tablets for three days (6, 4 and 3 mg) for swallowing difficulties and potassium supplements for the hypokalaemia noted in the blood tests (Table 1).

The patient was readmitted to the Infectious Diseases Department the next day (21/4/2020) (Fig. 1). Her COVID-19 symptoms (cough, myalgia, abdominal pain and fever) had worsened and she now presented with dyspnoea. At readmission, the patient’s respiratory rate 42 breaths/minute, blood pressure 114/61, pulse was 120 beats/minute and temperature 38.9 ^o^C. During episodes of coughing, her oxygen saturation fell to 86%, but with 5 L of oxygen on mask the saturation rose to 99%. Laboratory tests are shown in Table 1.

Her condition deteriorated on day 2 (22/4/2020) of the readmission. In addition to the generalized pain and tenderness, the pain in her right upper abdomen had worsened. Blood tests showed elevation of aspartate aminotransferase (ASAT), interleukin-6 (IL-6) and ferritin concentrations. There was impaired coagulation as shown by a prolonged activated partial thromboplastin time (APTT), high D-dimer, falling platelet count and decreased level of Anti-thrombin III (Table 1). Hemolysis was indicated by a fall in the hemoglobin concentration and rising lactate dehydrogenase levels although haptoglobin concentrations only were slightly elevated (Table 1). Despite her worsening condition, the patient felt active fetal movements and normal intermittent CTG controls were registered. Intravenous antibiotic treatment with Cefotaxime (2 g, 3 times daily) was initiated due to suspicion of concomitant bacterial infection (Table 1). Blood and urine cultures were taken but since the general condition of the patient had worsened, a decision was made to deliver by Caesarean section (32 + 6 gestational weeks), on maternal indication. The operation was performed in spinal analgesia in an operating theatre with negative air ventilation. The local hospital guidelines were followed to prevent the spread of COVID-19 [3]. An uncomplicated operation was completed within 40 min and the total blood loss was 200 millilitres (mL).

**Fig. 2:**
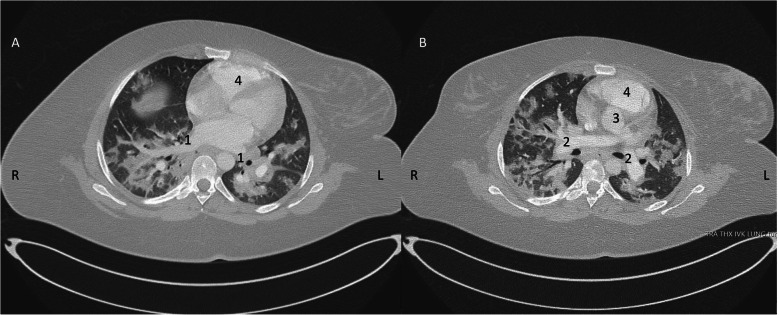
Computed tomography (CT) thorax scan showing bilateral diffuse, ground-glass opacities with both peripheral and perihilar distribution and no signs of pulmonary embolism. Two segments from the same session post-partum on day 2 of readmission (22/4/2020). 1 Pulmonary vein. 2 Pulmonary artery. 3 Aorta. 4 Right ventricle

After two hours in the post-operative unit, the patient returned to the ward and received thromboprophylaxis, dalteparin at a total dose of 10.000 units divided in two doses. A computed tomography (CT) lung scan, performed later the same day, showed bilateral diffuse, ground-glass opacities with both peripheral and perihilar distribution, but no signs of pulmonary embolism (Fig. 2).

### Post-operative pain management

Due to deranged liver values, the patient was unable to receive paracetamol and due to the COVID-19 infection not able to receive ibuprofen [4]. The pain relief was managed by administering 2.5 mL of intravenous morphine as needed. However, the patient’s condition worsened during the night and on examination, the patient was somnolent and lethargic but answered adequately when woken up (Reaction Level Scale 2). The patient’s pain was mostly localized to the upper right quadrant of the abdomen and epigastrium. Her uterus was well contracted and there were no signs of postoperative complications such as bleedings and local infection. On examination, the patient had miotic pupils that reacted poorly to light stimulation. Even though the patient only was given 7.5 mg of morphine over the course of 8 h, a morphine over-dose was suspected, and intravenous morphine was replaced by a combination of orally administered Naloxone and Oxycodone. Post-operative mobilization was initiated one day after surgery where after the patient made a steady recovery.

Table 1 illustrates the drastic improvement in the patient’s blood tests on day 3 (23/4/2020) of the readmission. The patient was discharged in good health on the 30th of April, 2020 with thromboprophylaxis planned for 6 weeks postpartum and a follow-up visit to the Obstetrics Clinic.

### The neonate

A male baby was delivered with birth weight 2085 g (37th percentile), birth length 48 cm (99th percentile) and head circumference 33.5 cm (99th percentile). The cord was clamped immediately after birth and the baby was shown briefly to the mother before being taken to a neonatal resuscitation station. At 1-minute after delivery, the baby had a normal heart rate but was gasping and had absent tone and no spontaneous movements. Positive pressure ventilation by a T-piece (neopuff) was given with peak inspiratory pressure (PIP) 20 cmH_2_O and positive end expiratory pressure (PEEP) between 5 and 7 cmH_2_O intermittently during the first seven minutes of life. At ten minutes, the baby was spontaneously breathing, albeit grunting, through T-piece continuous positive airway pressure (CPAP) with preductal oxygen saturation between 90–95% at FiO_2_ (fraction of inspired oxygen) 0.4. Fine crackles could be heard on lung auscultation. Intermittent intercostal retractions were also seen. Skin colour, tone and reflex irritability improved gradually during the stabilisation process. Apgar score; 1 min: 4 (appearance 1, pulse 2, reflex irritability 0, activity 1, respiration 0), 5 min: 6 (appearance 1, pulse 2, reflex irritability 1, activity 1, respiration 1), 10 min: 8 (appearance 2, pulse 2, reflex irritability 1, activity 1, respiration 2). Vitamin K was given intramuscularly and a nasogastric tube was inserted. Nasal CPAP with PEEP 6 cmH_2_O was started.

Arterial umbilical cord blood gas showed mild combined respiratory and metabolic acidosis with pH 7.28, partial pressure of oxygen (pO_2_) 3.45 (kilopascal) kPa, partial pressure of carbon dioxide (pCO_2_) 6.97 kPa and base excess − 6.8.

The baby was put in an incubator and transferred to the neonatal intensive care unit (NICU) and placed in an airborne infection isolation room (AIIR) with negative pressure ventilation. It was given preterm formula supplemented with intravenous glucose infusion. Venous blood gas was analysed at 4 h of age: pH 7.27, pO2 5 kPa, pCO2 7.3 kPa, BE -1.8, Hb 184 g/l and lactate 2.7 mmol/l.

After parental consent, formula was changed to donated breast milk. The mother was supplied with a breast pump and instructed in its use. After the initial need of breathing support and supplemental oxygen during day one of life the baby has enjoyed an uneventful clinical course. Nasopharyngeal swabs for SARS-CoV-2 detection were collected at 48 and 96 h of life and were found to be negative in both instances.

## Discussion and conclusions

We report a case of severe COVID-19 during in third trimester pregnancy, which led to an emergency Caesarean section and preterm delivery at 32 + 6 weeks of gestational age. This case adds to the growing body of evidence which raises concerns about the possible negative maternal outcomes of COVID-19 infection during pregnancy [1, 5–8]. Whilst most mothers with COVID-19 infection have mild symptoms which resolve without treatment, a number of cases have now emerged in the literature, where mothers have required intensive care admission, and one case requiring invasive ventilation with extracorporeal membrane oxygenation [8]. It has been suggested that pregnancy induced immune responses and cardio-vascular changes can exaggerate the course of the COVID-19 infection [1]. Mothers with prior medical conditions may be at higher risk for poor outcomes. In this case, there were several risk factors for preeclampsia such as Somalian origin and high BMI [9].

Our patient was severely obese (BMI 47 kg/m^2^) and her condition deteriorated drastically 12 days after her initial symptoms. Atypical HELLP (Haemolysis, Elevated Liver Enzymes, and Low Platelet Count) syndrome or Mississippi class 3 [10] can present itself with platelets between 100 and 150 × 10^9^/L, aspartate aminotransferase (ASAT)/alanine aminotransferase (ALT) ≥ 0.68 µkat/L and lactate dehydrogenase (LDH) > 10.2 µkat/L but it is seldom associated with coagulation impairment [10]. In addition, the patient had remarkably high levels of ASAT (28 µkat/L), as compared to the other liver tests, which is not consistent with the generalized liver dysfunction seen in HELLP or acute fatty liver of pregnancy [11].

Liver injury has been reported by a number of studies in patients with severe COVID-19 [12–15] making this organ the most commonly affected besides the respiratory system. Transient elevation of serum aminotransferases is often seen and a number of factors have been implicated for acute liver damage in severe COVID-19, including severe hypoxemia due to acute respiratory failure, drug interactions, septic shock and multiorgan dysfunction [12]. On readmission, the plasma paracetamol concentration was well within the therapeutic reference interval (Table 1) and liver injury secondary to a paracetamol overdose was therefore not suspected. Although there is insufficient evidence for direct SARS-CoV-2 virus-related hepatocyte injury, liver dysfunction has been continuingly related to severe COVID-19 infection [15] and intensive care admission [16, 17].

The patient also presented with elevated concentrations of several active-phase proteins (APPs) including IL-6, procalcitonin and ferritin, which may have indicated concomitant bacterial infection or severe systemic inflammation due to COVID-19. Subsequent blood and urine cultures were negative but the patient still received prophylactic treatment with broad spectrum antibiotics intravenously from day 1–5 of the readmission.

The hyperactive immune responses characteristic of severe COVID-19 can lead to stress-induced tissue injury and multiorgan impairment [18]. Ruan et al. [19] found that elevated levels of IL-6 were associated with a significantly increased risk of mortality in COVID-19 patients but in this case the inflammatory markers (and liver enzyme tests) decreased significantly after the Caesarean section. We can only speculate whether this indicated that severe COVID-19 in pregnancy may improve promptly by the termination of the pregnancy or that the patient’s condition was a combination of atypical HELLP and COVID-19 which subsequently improved after the Caesarean section. Considering the latter option, the patient showed some typical characteristics of HELLP syndrome; including right upper abdominal pain, epigastric pain, nausea and vomiting but a normal blood pressure [20]. Furthermore, she had no past obstetric history of preeclampsia or HELLP. Her platelet count was only marginally decreased and although subtle signs of haemolysis were present (decreased haemoglobin concentration and elevated LDH), haptoglobin concentration was not decreased [10] (Table 1). Given that pregnancy itself is a hypercoagulable state, it has been suggested that COVID-19 infection during pregnancy is associated with high risk of maternal thrombotic complications [15–17]. High levels of D-dimer in combination with elevated liver enzymes supports the likelihood that the patient’s liver injury and coagulation dysfunction were secondary to the severe COVID-19 infection.

Considering the neonate, we found no evidence for any vertical transmission of COVID-19 between mother and the baby. However, there have been reported cases of early COVID-19 detection in newborns, implying the potential risk of vertical transmission [21], although in the vast majority of cases, no such evidence has been identified [22, 23]. Since only a handful of cases have been reported in the literature, vertical transmission cannot be ruled out until systematic studies have been undertaken.

Our report has some limitations. We were unable to report a complete panel of coagulation tests due to errors in lab analysis. Similarly, the presence of antiphospholipid antibodies to rule out antiphospholipid syndrome were not investigated. We did not evaluate the presence of SARS-CoV-2 in amniotic fluid, cord blood, or placental tissue which could further clarify the possibility of vertical transmission.

In summary, we describe a severe case of maternal COVID-19 during the third trimester of pregnancy which led to liver and coagulation impairment and preterm delivery. We believe these findings have important public implications both due to the severity of the disease progression but also due to the rapid nature of the improvement after delivery. Atypical presentation of HELLP could not be ruled out and the importance of a multidisciplinary team in the treatment and management of severe COVID-19 during pregnancy is critical for positive patient outcome. Pregnant women should be considered a vulnerable group in the population in which exposure to COVID-19 should be avoided at all costs.

## Abbreviations

ASAT: Aspartate aminotransferase
BMI: Body mass index
CTG: Cardio-tocography
cm: Centimetre
CT: Computed tomography
COVID-19: Novel coronavirus 2019
HELLP: Hemolysis, Elevated Liver Enzymes and Low Platelet Count
Il-6: Interleukin-6
mg: Milligrams
SARS-CoV-2: Severe acute respiratory syndrome coronavirus 2

## Availability of data and materials

The datasets used and analysed during the current study available from the corresponding author on reasonable request.

## References

[1] Zaigham M, Andersson O. Maternal and perinatal outcomes with COVID-19: A systematic review of 108 pregnancies [published online ahead of print, 2020 Apr 7]. Acta Obstet Gynecol Scand. 2020. 10.1111/aogs.13867. 10.1111/aogs.13867 PMC7262097 32259279

[2] Liang H, Acharya G (2020). Novel corona virus disease (COVID-19) in pregnancy: What clinical recommendations to follow?. Acta Obstet Gynecol Scand.

[3] Coronavirus (COVID-19) Infection in Pregnancy. Information for healthcare professionals Version 7: Published Thursday 9 April 2020. Assessed May. 2nd, 2020. Available online: https://www.rcog.org.uk/globalassets/documents/guidelines/2020-04-09-coronavirus-covid-19-infection-in-pregnancy.pdf.

[4] Are patients with hypertension and diabetes mellitus at increased risk for COVID-19 infection? Fang L. Karakiulakis G, Roth M. Lancet Respir Med. 2020; [Epub ahead of print]. 10.1016/S2213-2600(20)30116-8 PMC7118626 32171062

[5] Schwartz DA. GrahamAL. Potentialmaternal and infant outcomesfrom (Wuhan) coronavirus 2019-nCoV infecting pregnant women: lessons from SARS, MERS, and other human coronavirus infections. Viruses. 2020;12(02):194. 10.3390/v12020194 PMC7077337 32050635

[6] Chen H, Guo J, Wang C (2020). Clinical characteristics and intrauterine vertical transmission potential of COVID-19 infection in nine pregnant women: a retrospective review of medical records. Lancet.

[7] Breslin N, Baptiste C, Miller R, et al. COVID-19 in pregnancy: early lessons. Am J Obstet Gynecol MFM. 2020;2(2):100111. [Epub ahead of print]. 10.1016/j.ajogmf.2020.100111 PMC7271091 32518902

[8] Liu Y, Chen H, Tang K, Guo Y (2020). Clinical manifestations and outcome of SARS-CoV-2 infection during pregnancy. J Infect.

[9] Ramos Amorim MM, Soligo Takemoto ML, Fonseca EB. Maternal Deaths with Covid19: a different outcome from mid to low resource countries? Am J Obstet Gynecol. 2020. [Epub ahead of print]. 10.1016/j.ajog.2020.04.023 PMC7195009 32348744

[10] Martin JN Jr, Rinehart BK, May WL, Magann EF, Terrone DA, Blake PG. The spectrum of severe preeclampsia: comparative analysis by HELLP (hemolysis, elevated liver enzyme levels, and low platelet count) syndrome classification. Am J Obstet Gynecol. 1999 Jun;180(6 Pt 1):1373–84. 10.1016/s0002-9378(99)70022-0 10368474

[11] Naoum EE, Leffert LR, Chitilian HV, Gray KJ, Bateman BT (2019). Acute Fatty Liver of Pregnancy: Pathophysiology, Anesthetic Implications, and Obstetrical Management. Anesthesiology.

[12] Li J, Fan JG (2020). Characteristics and Mechanism of Liver Injury in 2019 Coronavirus Disease. J Clin Transl Hepatol.

[13] Zu ZY, Jiang MD, Xu PP, Chen W, Ni QQ, Lu GM, et al. Coronavirus disease 2019 (COVID-19): A perspective from China. Radiology. 2020;296(2):E15–25. 10.1148/radiol.2020200490 PMC7233368 32083985

[14] Ren M, Jie L, Jun S, Subrata G, Liang-Ru Z, Hong Y, et al. Implications of COVID-19 for patients with pre-existing digestive diseases. Lancet Gastroenterol Hepatol. 2020. 10.1016/S2468-1253(20)30076-5 PMC7103943 32171057

[15] Guan WJ, Ni ZY, Hu Y, Liang WH, Ou CQ, He JX, et al. Clinical characteristics of coronavirus disease 2019 in China. N Engl J Med. 2020. 10.1056/NEJMoa2002032 PMC7092819 32109013

[16] Huang C, Wang Y, Li X, Ren L, Zhao J, Hu Y (2020). Clinical features of patients infected with 2019 novel coronavirus in Wuhan, China. Lancet.

[17] Wang D, Hu B, Hu C, Zhu F, Liu X, Zhang J (2020). Clinical characteristics of 138 hospitalized patients with 2019 novel coronavirus-infected pneumonia in Wuhan, China. JAMA.

[18] Henderson LA, Canna SW, Schulert GS, Volpi S, Lee PY, Kernan K, et al. On the alert for cytokine storm: Immunopathology in COVID-19. Arthritis Rheumatol. 2020. Accepted Author Manuscript. [Epub ahead of print]. 10.1002/art.41285 PMC7262347 32293098

[19] Ruan Q, Yang K, Wang W, Jiang L, Song J. Clinical predictors of mortality due to COVID-19 based on an analysis of data of 150 patients from Wuhan, China. Intens Care Med 2020 [Epub ahead of print]. 10.1007/s00134-020-05991-x PMC7080116 32125452

[20] Dusse LM, Alpoim PN, Silva JT, Rios DR, Brandão AH, Cabral AC (2015). Revisiting HELLP syndrome. Clin Chim Acta.

[21] Zhang ZJ, Yu XJ, Fu T, Liu Y, Jiang Y, Yang BX, Bi Y. Novel Coronavirus Infection in Newborn Babies Under 28 Days in China. Eur Respir J. 2020 Apr 8. pii: 2000697. [Epub ahead of print]. 10.1183/13993003.00697-2020 PMC7144267 32269087

[22] Yang P, Wang X, Liu P, Wei C, He B, Zheng J, Zhao D. Clinical characteristics and risk assessment of newborns born to mothers with COVID-19. J Clin Virol. 2020 Apr 10;127:104356. [Epub ahead of print]. 10.1016/j.jcv.2020.104356 PMC7194834 32302955

[23] Liu W, Wang J, Li W, Zhou Z, Liu S, Rong Z. Clinical characteristics of 19 neonates born to mothers with COVID-19. Front Med. 2020 Apr 13. [Epub ahead of print]. 10.1007/s11684-020-0772-y PMC7152620 32285380

